# Dosimetric advantages of O‐ring design radiotherapy system for skull‐base tumors

**DOI:** 10.1120/jacmp.v15i2.4608

**Published:** 2014-03-06

**Authors:** Kengo Ogura, Takashi Mizowaki, Yuichi Ishida, Masahiro Hiraoka

**Affiliations:** ^1^ Department of Radiation Oncology and Image‐applied Therapy Graduate School of Medicine Kyoto University Kyoto Japan

**Keywords:** dynamic conformal arc radiotherapy, dosimetric comparison, O‐ring design radiotherapy system, skull‐base tumors, fractionated stereotactic radiotherapy

## Abstract

The purpose of this study was to investigate whether a new O‐ring design radiotherapy delivery system has advantages in radiotherapy planning for skull‐base tumors. Twenty‐five patients with skull‐base tumors were included in this study. Two plans were made using conventional (Plan A) or new (Plan B) techniques. Plan A consisted of four dynamic conformal arcs (DCAs): two were horizontal, and the other two were from cranial directions. Plan B was created by converting horizontal arcs to those from caudal directions making use of the O‐ring design radiotherapy system. The micromultileaf collimators were fitted to cover at least 99% of the planning target volume with prescribed doses, 90% of the dose at the isocenter. The two plans were compared in terms of target homogeneity, conformity, and irradiated volume of normal tissues, using a two‐sided paired t‐test. For evaluation regarding target coverage, the homogeneity indices defined by the International Commission on Radiation Units and Measurements 83 were 0.099±0.010 (mean ± standard deviation) and 0.092±0.010, the conformity indices defined by the Radiation Therapy Oncology Group were 1.720±0.249 and 1.675±0.239, and the Paddick's conformity indices were 0.585±0.078 and 0.602±0.080, in Plans A and B, respectively. For evaluation of irradiated normal tissue, the Paddick's gradient indices were 3.118±0.283 and 2.938±0.263 in Plans A and B, respectively. All of these differences were statistically significant (p‐values <0.05). The mean doses of optic nerves, eyes, brainstem, and hippocampi were also significantly lower in Plan B. The DCA technique from caudal directions using the new O‐ring design radiotherapy system can improve target homogeneity and conformity compared with conventional DCA techniques, and can also decrease the volume of surrounding normal tissues that receives moderate doses.

PACS numbers: 87.55.‐x, 87.55.D‐, 87.55.dk

## I. INTRODUCTION

Fractionated stereotactic radiotherapy (FSRT) is a sophisticated technique for intracranial tumors. For large skull‐base tumors, such as pituitary adenoma (macroadenoma) and sellar or parasellar meningioma, definitive surgical approaches are sometimes difficult because of surrounding eloquent structures, and FSRT is well indicated for such cases. The dynamic conformal arc (DCA) technique is a useful approach in FSRT.^(1)^ This has become available with the introduction of multileaf collimators (MLCs). The shape of the radiation field in each beam's eye view is fitted to the target volume throughout the arc length, which yields high dose conformity to the targets.

In conventional C‐arm linear accelerator (linac) systems, arc arrangement from caudal directions is usually difficult because of the risk of collision between the gantry head and the patient. For small tumors, arcs from only horizontal and cranial directions would be sufficient to achieve high dose homogeneity to the target. However, for large tumors, these are occasionally insufficient because the dose distribution within the targets becomes inhomogeneous from the cranial to caudal direction, such that fewer doses are delivered to the caudal part of the target. In this regard, we hypothesized that the characteristics of a new O‐ring design radiotherapy system, vero4DRT (Mitsubishi Heavy Industries, Ltd., Tokyo, Japan, and BrainLAB, Feldkirchen, Germany) would yield further improvements in target conformity and homogeneity, compared with conventional DCA techniques.

**Figure 1:**
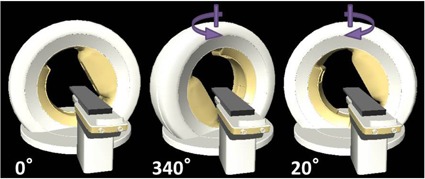
External appearance of O‐ring rotation. The O‐ring can be rotated around its vertical axis without any couch movement (indicated by arrows). Examples of rotation angles of 340° and 20° (counterclockwise or clockwise), are shown.

The vero4DRT has a unique configuration. The details are described elsewhere.^2^, ^3^ In brief, a 6 megavolt ultracompact and light‐weight C‐band linac with 5 mm width MLCs are mounted on the inside of a rigid O‐ring‐shaped gantry and is designed to rotate 360° along the inner surface of the O‐ring. Additionally, the O‐ring gantry, per se, can be rotated $\pm 60^{\circ }$ around the vertical axis of the O‐ring. This enables delivery of noncoplanar beams from both cranial and caudal directions without movement of the treatment couch (Fig. 1). In addition, electronic portal‐imaging devices, two sets of kilovoltage (kV) X‐ray tubes and flat‐panel detectors, and a kV‐level cone‐beam computed tomography (CT) platform, are mounted on the gantry. The treatment couch can translate right‐to‐left, superior‐to‐posterior, and anterior‐to‐posterior, and can also rotate in both the pitch and roll planes. Use of such tools allows of high‐precision, image‐guided patient setup.^(4)^ The MLC of the delivery systems is regular in type, as opposed to a binary MLC (such as employed in tomotherapy). The treatment couch does not translate or rotate during irradiation. The MLC is of single‐focus, has 30 pairs of 5 mm wide leaves at the isocenter, and produces a maximum treatment field of $150\times 150\;{\text{mm}}^{2}$.^(5)^

The aim of this study was to investigate whether the new O‐ring design radiotherapy system, vero4DRT, has advantages in radiotherapy planning for intracranial tumors. We performed a dosimetric comparison of treatment planning with and without DCAs from caudal directions for large skull‐base tumors.

## II. MATERIALS AND METHODS

## A. Tumor characteristics

Twenty‐five patients with skull‐base tumors (11 pituitary adenomas, eight meningiomas, and six craniopharyngiomas) were included in this replanning study. All had large tumors, which were difficult to resect completely, and previously underwent conventional FSRT (50.4−54 Gy in 28−30 fractions). We chose sellar or parasellar skull‐base tumors of various sizes and shapes, and excluded extremely irregular tumors, which were treated with intensity‐modulated radiotherapy. The median planning target volume (PTV) was 23.16 cc (range, 7.10−68.71).

## B. Treatment planning

Treatment planning was conducted using previously acquired computed tomography images and the iPlan RT Dose planning system, version 4.5.1 (BrainLAB). The CT images of 1.25 mm slice thickness were acquired using a Light Speed RT scanner (GE Healthcare, Milwaukee, WI). Patients were immobilized in a thermoplastic mask with an additional bite block and infrared reflecting markers (BrainLAB) on the surface of the mask. Contrast‐enhanced magnetic resonance imaging (MRI) scans were fused with planning CT images using iPlan RT Image version 4.1.2 (BrainLAB). Clinical target volume (CTV) was defined as residual gross tumors — except cases of craniopharyngioma, in which the CTV was defined as residual gross tumors and 5 mm thick areas of the normal brain tissue attached to the tumors on preoperative MRI images. Then, the PTV was defined as the CTV plus a 2 mm margin, to account for setup errors and patient motion. In addition, lenses, eyes, optic nerves, optic chiasm, brainstem, temporal lobes, hippocampi, cochleae, and parotid glands were contoured as organs at risk (OARs). The hippocampal contouring was based on the report by Gondi et al.^(6)^

**Figure 2:**
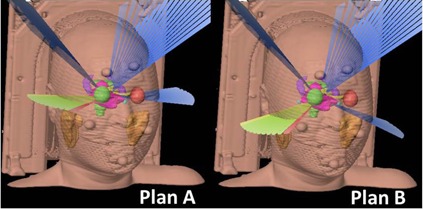
Three‐dimensional views of the conventional and new treatment planning. A representative case of pituitary adenoma is shown. A planning target volume is indicated in magenta. Plan A is a conventional arrangement composed of four dynamic conformal arcs, two horizontal arcs and the remaining two from cranial directions. Plan B is a new method, which includes two arcs from caudal directions.

Two plans were made using conventional (Plan A) and new (Plan B) techniques, as shown in Fig. 2. Both plans were made using the same treatment planning systems (vero4DRT and iPlan) to exclude the effects of all parameters other than arc direction. Plan A was a conventional plan produced using the vero4DRT and iPlan systems. Plan A consisted of four DCAs. Two were horizontal arcs, or the O‐ring rotation angles were 0°. The other two arcs were from cranial directions, or the O‐ring rotation angles were set to $40^{\circ }-45^{\circ }$ or $315^{\circ }-320^{\circ }$. This arc arrangement is typical in our daily clinical practice and is also similar to that used for DCA plans in the literature.^(1)^ Vulnerable organs such as lenses were avoided as much as possible by adjusting the length of each arc. Next, the MLCs were fitted to cover at least 99% of the planning target volume (PTV) with the prescribed dose, which was 90% of the dose at the isocenter. To simplify the comparison between the new technique (Plan B) and the conventional planning (Plan A), we modified Plan A by converting only horizontal arcs to those from caudal directions (Plan B). The rotation angles of the O‐ring were set to $15^{\circ }-20^{\circ }$ or $340^{\circ }-345^{\circ }$, to take into account the interference between the patients/couch and the gantry head/O‐ring itself. The lengths of arcs were also modified if lenses were included in the beam's eye view of the targets.

### C. Dosimetric comparison

The two techniques were compared in terms of target homogeneity, target conformity and irradiated volume of normal tissues. The dose calculations were performed using the radiological path length method for tissue heterogeneity correction. The grid size of the dose volume histogram (DVH) calculation was set to 2.0 mm.

Target homogeneity was quantified by the homogeneity index (HI) defined in Report 83 of the International Commission on Radiation Units and Measurements (ICRU) as $\text{HI}=(D_{2\%}-D_{98\%})/D_{50\%}$, where $D_{2\%},D_{98\%}$, and $D_{50\%}$ are the doses covering at least 2%, 98%, and 50% of the PTV, respectively. Target conformity was quantified using the conformity index (CI) defined by the Radiation Therapy Oncology Group (${\text{CI}}_{\text{RTOG}}$)^(7)^ as: ${\text{CI}}_{\text{RTOG}}=\text{PIV}/\text{TV}$, where PIV is the whole tissue volume receiving the prescribed dose (90% of the dose at the isocenter) and TV is volume of PTV. In addition, target conformity was evaluated by the index described by Paddick et al. (${\text{CI}}_{\text{Paddick}}$)^(8)^ as: ${\text{CI}}_{\text{Paddick}}={V_{\text{PTV}\_\text{PI}}}^{2}/(\text{PIV}\times \text{TV})$, where $V_{\text{PTV}\_\text{PI}}$ is the volume of PTV receiving at least the prescribed dose. Irradiated volume of normal tissue and dose gradient were analyzed by comparing the isodose volumes (IDVs), known as Paddick's gradient index (PGI):^(9)^ PGI=50%/100%IDVs, where the prescribed dose was normalized to 100%IDV. For evaluation of the normal tissue volume receiving much lower doses, modified PGI (mPGI) was also defined as: mPGI=20%/100%IDVs.

### D. Statistical analysis

For statistical analysis, we used the statistical software environment R, version 2.15.0 (http://www.r-project.org). To confirm the null hypothesis that the differences between Plan A and Plan B parameters were zero, we applied the two‐sided paired t‐test. P‐values <0.05 were considered to indicate statistical significance.

## III. RESULTS

**Figure 3:**
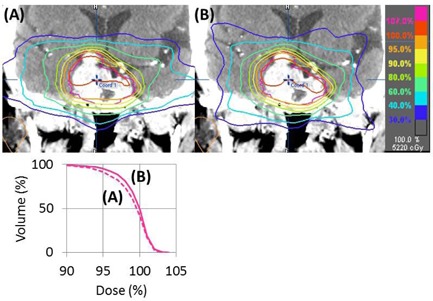
A representative case in which target coverage was improved. Coronal images of dose distributions in Plans A and B and the dose‐volume histogram are shown. Target coverage of the caudal part was improved in Plan B (see 95% isodose line indicated as an orange line). The dose‐volume histogram of planning target volume also shows the improvement in Plan B. In addition, the volume of the bilateral temporal lobes that received moderate doses was decreased in Plan B.

Table 1 summarizes the result of each index (mean±standard deviation(SD)). All indices of target homogeneity and conformity (HI, ${\text{CI}}_{\text{RTOG}}$, and ${\text{CI}}_{\text{Paddick}}$) in Plan B were improved significantly. A representative case is shown in Fig. 3. In terms of irradiated normal tissues, PGI decreased and mPGI increased in Plan B, both with statistical significance. Thus the volumes of normal tissues irradiated with a moderate dose (50% of the prescribed isodoses) were significantly decreased in Plan B, whereas the volumes with much lower doses (20% of the prescribed isodose) were significantly increased in Plan B.

Table 2 summarizes the mean and/or max dose of OARs (%). The dose at the isocenter was normalized to 100%. The mean doses of optic nerves, eyes, brainstem, and hippocampi, and the maximum doses of eyes were significantly decreased in Plan B. In contrast, the mean doses of both parotid glands were significantly increased in Plan B.

**Table 1:** Summary of indices in Plans A and B

	*Plan A*	*Plan B*	*Mean Difference*	
	*(conventional)*	*(new)*	*Plan B − Plan A*	
	Mean±SD(range)		*(95% CI)*	*p‐value*
HI	0.099±0.010	0.092±0.010	−0.007	<0.001
	(0.084−0.119)	(0.069−0.106)	(−0.011 to −0.004)	
${\text{CI}}_{\text{RTOG}}$	1.720±0.249	1.675±0.240	−0.045	0.005
	(1.353−2.339)	(1.313−2.334)	(−0.075 to −0.015)	
${\text{CI}}_{\text{Paddick}}$	0.585±0.078	0.602±0.080	+0.016	<0.001
	(0.424−0.732)	(0.423−0.751)	(+0.008 to +0.025)	
PGI	3.118±0.283	2.938±0.263	−0.179	<0.001
	(2.618‐3.737)	(2.566−3.502)	(−0.221 to −0.137)	
mP GI	11.533±1.629	12.254±1.410	+0.720	<0.001
	(8.238−14.845)	(9.573−14.845)	(+0.530 to +0.910)	

${\text{CI}}_{\text{Paddick}}=$ conformity index defined by Paddick *et al*.; ${\text{CI}}_{\text{RTOG}}=\text{CI}$ defined by the Radiation Therapy Oncology Group (RTOG); HI=homogeneity index; mPGI=modified PGI; $\text{PGI}={\text{Paddick}}^{\prime }\text{s gradient index}$; SD=standard deviation.

**Table 2:** Summary of the maximum and mean doses to organs at risk

	*Plan A*	*Plan B*	*Mean Difference*	
	*(conventional)*	*(new)*	*Plan B − Plan A*	
	Mean±SD(range)(%) ^a^		*(95% CI)*	*p‐value*
Rt. OPN	85.3±27.4	84.9±28.0	−0.47	
(max. dose)	(13.1−103.2)	(14.4−102.4)	(−1.32 to +0.38)	0.266
Rt. OPN	34.8±17.6	33.0±17.6	−1.79	
(mean dose)	(5.1−67.5)	(4.8−67.7)	(−2.51 to −1.07)	<0.001
Lt. OPN	88.1±27.8	87.6±29.2	−0.54	
(max. dose)	(8.6−103.0)	(8.7−102.5)	(−1.34 to −0.26)	0.176
Lt. OPN	35.4±17.3	33.4±17.0	−2.01	
(mean dose)	(2.8−61.0)	(2.8−61.1)	(−2.88 to −1.15)	<0.001
Rt. Eye	7.9±3.4	6.1±2.7	−1.74	
(max. dose)	(1.2−14.5)	(1.2−11.7)	(−2.51 to −0.96)	<0.001
Rt. Eye	2.1±0.9	1.8±0.7	−0.25	
(mean dose)	(0.9−3.9)	(0.8−3.7)	(−0.38 to −0.13)	<0.001
Lt. Eye	9.2±4.7	7.7±4.6	−1.52	
(max. dose)	(4.1−23.0)	(2.3−24.3)	(−2.19 to −0.85)	<0.001
Lt. Eye	2.4±1.1	−0.30	(0.9−4.9)	
(mean dose)	(0.9−4.9)	(0.8−4.8)	(−0.43 to −0.18)	<0.001
Chiasm	93.5±19.0	93.4±18.5	−0.04	
(max. dose)	(17.7−103.7)	(20.9−103.1)	(−0.62 to +0.53)	0.876
Chiasm	83.8±26.2	83.6±26.1	−0.20	
(mean dose)	(11.3−103.0)	(12.4−102.1)	(−1.20 to +0.80)	0.684
Brainstem	36.0±14.8	35.4±15.0	−0.60	
(mean dose)	(13.2−66.3)	(12.9−64.3)	(−1.09 to −0.11)	0.020
Rt. PG	6.5±2.9	8.7±4.5	+2.20	
(mean dose)	(2.6−16.5)	(2.6−25.2)	(+1.40 to +3.00)	<0.001
Lt. PG	6.3±2.3	8.8±3.8	+2.52	
(mean dose)	(2.6−11.6)	(2.6−18.5)	(+1.62 to +3.43)	<0.001
Rt. cochlea	42.0±24.2	43.0±23.2	+0.98	
(max. dose)	(12.0−95.5)	(18.9−94.7)	(−0.74 to +2.69)	0.251
Lt. cochlea	41.1±23.2	41.8±22.1	+0.76	
(max. dose)	(11.8−96.3)	(17.8−97.0)	(−1.08 to +2.61)	0.402
Rt. Hippo	32.8±10.4	30.4±10.7	−2.37	
(mean dose)	(12.0−48.7)	(11.2−47.4)	(−3.32 to −1.43)	<0.001
Lt. Hippo	35.5±16.3	33.5±16.7	−2.01	
(mean dose)	(7.6−73.9)	(6.8−74.4)	(−3.12 to −0.90)	0.001

^a^ Relative doses are presented (The dose distribution calculation was normalized to 100% at the isocenter). Hippo=hippocampus; Lt.=left; OPN=optic nerve; PG=parotid gland; Rt.=right.

## IV. DISCUSSION

In this study, we demonstrated the advantages of DCA techniques with the vero4DRT in terms of both target conformity and homogeneity. Beams/arcs from caudal directions can compensate for dose deficiency at the caudal parts of large skull‐base tumors, which would be underdosed areas if beams/arcs from only cranial directions could be available. The change in the absolute value of these indices might be a little, but improved with statistically significant levels. We believe that this tendency is a unique characteristic and a mechanical advantage of the O‐ring design radiotherapy system, vero4DRT. In addition, moderate doses to normal tissue were decreased in Plan B, whereas lower doses were increased. We used PGI/mPGI to evaluate these ranges of doses. Moderate and lower doses in our study corresponded to around 25 Gy and 10 Gy, respectively. Increases in moderate doses in Plan A should be due to opposed horizontal arcs, whereas increases in lower doses in Plan B should be due to the caudal arcs which resulted in more irradiated volume. The life expectancy of patients with benign brain tumors is thought to be long and so the risk of second malignancy must be considered. Few data regarding whether a decrease in normal tissue with moderate doses and an increase in that with lower doses are clinically preferable are available. Galloway et al.^(10)^ reported the site of second tumor formation after radiotherapy to the central nervous system; the most common location of the second tumors was in tissue that received a moderate dose (20−36 Gy). They concluded that a decrease in the brain volume that receives a moderate radiation dose is the most important factor. We also showed that the DCAs technique with the vero4DRT results in a decrease in the tissue volume receiving a moderate dose, compared with the conventional techniques.

**Figure 4:**
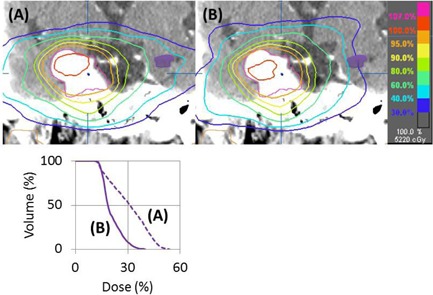
A representative case in which the irradiation dose to the left hippocampus was decreased. Coronal images of dose distributions in Plans A and B are shown. The irradiation dose received by the left hippocampus (purple shaded area) was decreased in Plan B. The dose volume histogram clearly shows the difference between Plans A and B.

The irradiated doses to OARs in Plans A and B were both within conventionally tolerated limits, according to the report of Emami et al.^(11)^ or Quantitative Analysis of Normal Tissue Effects in the Clinic (QUANTEC).^(12)^ Plan B has an advantage in decreasing doses to hippocampi, whereas it has a tendency to increase doses to parotid glands. When treating skull‐base tumors, beam entry of caudal arcs often runs through ipsilateral parotid glands and should result in significant increases of doses to them. On the other hand, caudal arcs should decrease irradiated doses to temporal lobes, whereas opposing horizontal arcs should increase the doses. In both cases, irradiated doses to temporal lobes are within tolerable limits in terms of radiation necrosis, but may lead to neurocognitive dysfunction. Some investigators have reported cognitive decline after definitive radiotherapy, and a correlation between cognitive decline and dose to the temporal lobes.^13^, ^14^, ^15^ Recently, the hippocampus has been reported to be a key component with regard to neurocognitive deficits after cranial irradiation.^(16)^ The relationships between the irradiation doses delivered to the hippocampus and the occurrence of neurocognitive dysfunction remains to be elucidated; however, some studies have reported that much lower doses can lead to neurological sequelae.^(17)^ Considering the long clinical courses of benign brain tumors, decreasing the doses to the hippocampi might be preferable. A representative case, in which the dose to the left hippocampus was decreased, is shown in Fig. 4.

In daily clinical practice, the vero4DRT would yield high patient throughput and be more comfortable because it can deliver noncoplanar arcs without any couch/patient movement. In conventional C‐arm linacs, radiotherapists or staff members are required to enter the treatment room for each couch rotation; however, this is not the case for the vero4DRT, and the O‐ring gantry can rotate and enable delivery of noncoplanar beams without any couch movement. Accordingly, the treatment time required is expected to be less. This is also preferable and more comfortable for the treated patient.

The vero4DRT has some mechanical limitations. First, the MLC width of vero4DRT is not very thin (5 mm), and the collimator cannot be rotated. Accordingly, the MLCs cannot be fully adjusted to concave and/or irregularly shaped tumors. We are at present developing a new model with 2.5 mm wide MLCs. Several reports show the impact of MLC width on target conformity and sparing of surrounding tissue.^18^, ^19^ The version in development will achieve more conformal planning for irregularly shaped tumors. Second, DCAs from cranial directions are not fully available using the vero4DRT compared with conventional C‐arm linacs, which can deliver even vertex arcs $\left(\text{couch angle}=90^{\circ }\right)$. Rotational angles of the O‐ring around the vertical axis are mechanically limited to $\pm 60^{\circ }$, and in the clinical setting $\pm 45^{\circ }-50^{\circ }$ O‐ring angles are limiting when using DCAs from cranial directions. This range of motion seems to be narrow, but large angular arcs, such as vertex arcs, should be avoided because the beams run through the full length of the body. In addition, DCAs from caudal directions would be able to compensate for these limitations in target coverage. Third, collision between the gantry head/O‐ring and the patients/couch would likely be an issue for tumors located at other sites in the brain, such as more anterior or lateral sides, whereas sellar or parasellar tumors are located close to the center of the brain. In this report, we discussed using DCAs from caudal directions for skull‐based tumors located in the sellar or parasellar areas, and that O‐ring rotational angles of $15^{\circ }-20^{\circ }$ or $340^{\circ }-345^{\circ }$ are limiting. An improved version of the iPlan is now under development; it will support a detailed collision map between the X‐ray head unit or the O‐ring and the couch or patient, which will enable safe delivery of noncoplanar DCAs.

In daily practice, we generally do not use caudal arcs when employing C‐arm conventional linacs because of the risk of collision between the gantry and the patient. However, conventional linacs allow caudal arcs to be used to some extent, although the available geometric freedom is less than that of the vero4DRT. We used a conventional linac (the Clinac iX; Varian Medical Systems, Palo Alto, CA) to experimentally explore how much angle could safely be assumed in the context of caudal arcs, In agreement with the literature,^(20)^ the range was maximally 5°. Next, we replanned three representative plans within this range of angle (the plans were termed Plans C). Compared to Plan A, the homogeneity indices decreased (thus improved) by −0.007,−0.0002, and −0.007, although the improvements tended to be smaller than those available under Plan B (−0.017,−0.019, and −0.010, respectively). In addition, the numbers of moderate doses delivered to normal tissue (the PGIs) also fell (thus improved) by −0.020,−0.226, and −0.097 when Plan C was implemented, but the improvements under Plan B were again better (−0.206,−0.361, and −0.397, respectively). Thus, even the small‐range (5°) caudal arcs accessible using conventional linacs can improve target homogeneity and decrease doses delivered to normal tissues.

## IV. CONCLUSIONS

DCA techniques from caudal directions using the vero4DRT can improve target homogeneity and conformity for skull‐base tumors, compared with conventional DCA techniques. Moreover, the volume of surrounding normal tissue that receives a moderate dose is also reduced. We believe that this is one of the defining characteristics of the vero4DRT. The clinical impact of this new technique is a separate question that will be addressed in future studies.

## ACKNOWLEDGMENTS

This research was funded by a grant from the Japan Society for the Promotion of Science (JSPS) through the “Funding Program for World‐Leading Innovative R&D on Science and Technology (FIRST Program)”, initiated by the Council for Science and Technology Policy (CSTP). T.M. and M.H. have a consultancy agreement with Mitsubishi Heavy Industries Ltd., Japan.

## Supporting information

Supplementary Material
