# Supplementary material for: Dosimetric advantages of O‐ring design radiotherapy system for skull‐base tumors
Source: J Appl Clin Med Phys. 2014 Mar 6;15(2):226–34. doi: 10.1120/jacmp.v15i2.4608 (PMC5875486; doi:10.1120/jacmp.v15i2.4608)
Supplement: Supplementary file 1 — Supplementary Material [file ACM2-15-226-s001.doc]

Dosimetric advantages of O-ring design radiotherapy system for skull-base tumors

**Kengo Ogura, M.D.**

**Takashi Mizowaki, M.D., Ph.D.**

**Yuichi Ishida, M.D.**

**Masahiro Hiraoka, M.D., Ph.D.**

*Department of Radiation Oncology and Image-applied Therapy*

*Graduate School of Medicine Kyoto University*

*54 Kawahara-cho Shogoin Sakyo-ku, Kyoto 6068507, Japan*

[*mizo@kuhp.kyoto-u.ac.jp*](mailto:mizo@kuhp.kyoto-u.ac.jp)

Corresponding author: Takashi Mizowaki, Department of Radiation Oncology and Image-applied Therapy, Graduate School of Medicine Kyoto University, 54 Kawahara-cho Shogoin Sakyo-ku, Kyoto 6068507, Japan; phone +81-75-751-3762; fax: +81-75-751-3419; email: [mizo@kuhp.kyoto-u.ac.jp](mailto:mizo@kuhp.kyoto-u.ac.jp)

Running title

O-ring design RT system
